# The Arabic Patient-Specific Functional Scale Demonstrates Responsiveness in Detecting Changes in Lower Extremity Function Among Individuals with Lower Extremity Musculoskeletal Disorders

**DOI:** 10.3390/jcm15156009

**Published:** 2026-08-02

**Authors:** Mishal M. Aldaihan, Abdulrahman M. Alsubiheen, Ali H. Alnahdi

**Affiliations:** 1 Department of Rehabilitation Sciences, College of Applied Medical Sciences, King Saud University, P.O. Box 10219, Riyadh 11433, Saudi Arabia; mishaldaihan@ksu.edu.sa (M.M.A.);; 2 King Salman Center for Disability Research, Riyadh 11614, Saudi Arabia

**Keywords:** psychometrics, sensitivity to change, patient-centered outcome, patient-reported outcome measures, lower extremity function

## Abstract

**Background/Objective:** The Patient-Specific Functional Scale (PSFS) is a patient-centered outcome measure that captures limitations in activities that are personally meaningful to patients. Although the Arabic PSFS has demonstrated acceptable validity and reliability, its responsiveness in individuals with lower extremity musculoskeletal disorders has not been established. This study aimed to examine the responsiveness of the Arabic PSFS in detecting changes in lower extremity function over time. **Methods:** A prospective cohort study was conducted in three outpatient physical therapy clinics in Riyadh, Saudi Arabia. Seventy-two adults with lower extremity musculoskeletal disorders receiving physical therapy care completed the Arabic PSFS, RAND-36, and Numeric Pain Rating Scale (NPRS) at baseline and follow-up. At the follow-up, participants also completed the Global Rating of Change (GRC). Responsiveness was evaluated using eight a priori hypotheses following COSMIN recommendations. Change scores were analyzed using correlation coefficients, paired *t*-tests, effect size (ES), and standardized response mean (SRM). **Results:** Sixty participants (83.3%) reported improvement in the GRC. PSFS scores improved significantly (mean difference = 1.66; *p* < 0.001), demonstrating a large magnitude of improvement (ES = 0.80; SRM = 0.92). PSFS change scores correlated moderately with RAND-36 physical functioning (r = 0.51) and GRC (r = 0.55) and weakly with emotional well-being (r = 0.26) and showed smaller-than-expected associations with pain measures. Six out of eight (75%) predefined hypotheses were confirmed. **Conclusions:** The Arabic PSFS demonstrates sufficient responsiveness in detecting improvement in lower extremity function and is suitable for monitoring rehabilitation outcomes in Arabic-speaking adults with lower extremity musculoskeletal disorders receiving outpatient physical therapy.

## 1. Introduction

Patient-centered care has emerged as the prevailing paradigm for musculoskeletal rehabilitation, underscoring the importance of individualized assessment and treatment strategies that are informed by patients’ preferences, aspirations, and intrinsic values [1,2,3]. Within this framework of care, the identification and quantification of outcomes that genuinely resonate with patients—such as their perceived capacity to engage in meaningful daily activities—has become imperative for refining treatment decisions and appraising the efficacy of care delivery [2,4]. Patient-reported outcome measures (PROMs) constitute a fundamental element of patient-centered practice, as they encapsulate the individual’s perspective on health, functional capabilities, and progression, rather than relying exclusively on clinician-observed performance metrics [5].

Among individuals with lower extremity musculoskeletal disorders, a variety of PROMs are extensively utilized to assess physical function, including the Lower Extremity Functional Scale, the Western Ontario and McMaster Universities Osteoarthritis Index, and the Hip Disability and Osteoarthritis Outcome Score [6,7,8]. Although these instruments possess evidence supporting their measurement properties, they comprise predetermined items established by developers within specific cultural and linguistic frameworks. Thus, they may neglect to account for activities that hold cultural or contextual significance for diverse populations [9], such as Arabic-speaking communities. This limitation accentuates the necessity for measures that can dynamically capture patient-relevant functional activities across varied cultural landscapes.

The Patient-Specific Functional Scale (PSFS) is an individualized PROM designed to overcome the limitations associated with fixed-item questionnaires [10]. Within the PSFS framework, patients articulate the activities they encounter difficulties with due to their condition and subsequently evaluate their ability in executing these tasks. The PSFS is inherently patient-centered and flexible, enabling it to encompass activity limitations and participation restrictions that are salient to each individual [11]. Its desirable attributes—conciseness, ease of administration, and clinical interpretability—have significantly contributed to its widespread adoption in the realm of musculoskeletal rehabilitation [12,13,14]. The existing body of evidence corroborates its measurement properties among patients presenting with various lower extremity musculoskeletal disorders [14].

The PSFS has undergone adaptation into the Arabic language utilizing rigorous methodological approaches and has exhibited satisfactory validity, test–retest reliability, and acceptable measurement error in individuals with lower extremity musculoskeletal disorders [15]. Nevertheless, to date, no investigation has been conducted to evaluate its responsiveness in Arabic-speaking patients suffering from lower extremity musculoskeletal disorders. According to the COnsensus-based Standards for the selection of health Measurement Instruments (COSMIN) framework, responsiveness is defined as “the ability of an instrument to detect change over time in the construct to be measured” [16]. Establishing responsiveness is crucial for validating the longitudinal integrity of the Arabic PSFS in individuals with lower extremity musculoskeletal disorders and for assessing whether score changes signify genuine enhancements in lower extremity function, as opposed to random fluctuations or measurement error [17]. In the absence of such evidence, the interpretability and clinical applicability of the Arabic PSFS in assessing lower extremity functional recovery remain ambiguous. Therefore, the aim of this study was to examine the responsiveness of the Arabic PSFS in detecting changes in lower extremity function over time among individuals with lower extremity musculoskeletal disorders. We hypothesized that the Arabic PSFS would be a responsive measure capable of validly capturing longitudinal changes in lower extremity function among individuals with lower extremity musculoskeletal disorders.

## 2. Materials and Methods

### 2.1. Study Design

This study employed a prospective cohort design to examine the responsiveness of the Arabic PSFS in detecting changes in lower extremity function over time among individuals with lower extremity musculoskeletal disorders.

### 2.2. Setting and Participants

Participants were recruited using convenience sampling from three outpatient physical therapy clinics in Riyadh, Saudi Arabia—namely, King Fahad Medical City, King Saud University Medical City, and Physiotrio Clinic—between November 2023 and October 2024. Ethical approval for the study was obtained from the Institutional Review Boards at the participating institutes (approval numbers: E-23-8145; 24-191E). All participants were provided with detailed information regarding the purpose, procedures, and requirements of the study. Written informed consent was obtained from each participant before data collection. Participants were eligible for inclusion if they were 18 years of age or older and had a primary complaint of a lower extremity musculoskeletal disorder and referred to physical therapy for management. Participants were excluded if they were unable to read or comprehend Arabic or reported any neurological, cardiovascular, pulmonary, or spinal conditions that negatively influenced their functional activities.

### 2.3. Procedure

The data were collected through two assessment sessions: an initial (baseline) evaluation and a follow-up evaluation. The baseline assessment was conducted during each participant’s first visit to the outpatient physical therapy clinic, while the follow-up assessment occurred during a later scheduled visit after a period of physical therapy intervention. Participants completed the follow-up assessment during their scheduled reassessment or discharge visit after receiving routine physical therapy. Between the two assessments, participants received individualized physical therapy according to the clinical judgment of their treating physical therapists. Treatment was not standardized because the objective was to evaluate the responsiveness of the Arabic PSFS under routine clinical practice rather than the effectiveness of a specific intervention. The study investigators did not influence or standardize the treatment protocols. Maintaining natural clinical variability was essential to reflect real-world practice conditions.

At baseline, participants completed the following outcome measures: the Arabic PSFS [15], RAND 36-Item Health Survey [18] and the Numeric Pain Rating Scale [19]. At the follow-up assessment, participants completed the same set of measures along with the Global Rating of Change scale (GRC) [20,21], which was used as an external anchor to determine perceived changes in function. All measures were completed during in-person sessions. The PSFS was clinician-administered using the standardized baseline and follow-up instructions, whereas the remaining questionnaires were self-administered.

### 2.4. Outcome Measures

#### 2.4.1. Patient-Specific Functional Scale (PSFS)

The Arabic PSFS was administered by the treating physical therapist using the standardized instructions of the original PSFS [10]. At baseline, participants were asked to identify three to five important activities that they were unable to perform or had difficulty performing because of their lower-extremity musculoskeletal disorder. The clinician recorded each nominated activity, and participants rated their current ability to perform each activity on an 11-point numerical scale ranging from 0 (“unable to perform the activity”) to 10 (“able to perform the activity at the pre-injury level”). At follow-up, the treating physical therapist referred to the previous assessment and read the same activities recorded at baseline to the participant, who then re-rated their current ability to perform each activity. The treating physical therapist, but not the participant, had access to the baseline recorded activities and ratings. The follow-up score was therefore based on the same patient-nominated activities assessed at baseline; no activities were added, removed, or replaced during the follow-up. The overall PSFS score was calculated as the mean of the activity ratings, yielding a score from 0 to 10, with higher scores indicating better functional ability. The administration and scoring procedures were consistent with those used in the validated Arabic version of the PSFS [15].

#### 2.4.2. RAND 36-Item Health Survey (RAND-36)

The RAND-36 is a widely used generic measure of health-related quality of life that assesses an individual’s perceived physical and mental health status [22]. It consists of 36 items grouped into eight domains, each reflecting a specific dimension of health. Scores for each domain are transformed to range from 0 to 100, with higher scores representing better health or functioning. The domains are physical functioning, bodily pain, role limitations due to physical health problems, role limitations due to personal or emotional problems, emotional well-being, social functioning, energy/fatigue, and general health perceptions. The Arabic version of the RAND-36 has been cross-culturally adapted and validated, demonstrating acceptable reliability and validity [18]. The RAND-36 was chosen in the current study because it measures domains similar and different to that measured by the PSFS, thus allowing the generation of convergent and divergent correlation hypotheses. Additionally, a lower extremity-specific Arabic comparator with established longitudinal measurement properties was not available when the study was designed.

#### 2.4.3. Numeric Pain Rating Scale (NPRS)

Pain intensity was measured using the NPRS, on which participants rated their average level of pain in the affected lower extremity region during the past 24 h [23]. Scores range from 0 (no pain) to 10 (worst imaginable pain). The Arabic NPRS has demonstrated good reliability and validity [19].

#### 2.4.4. Global Rating of Change Scale (GRC)

At the follow-up assessment, participants completed the GRC to indicate their perceived overall change in lower extremity function since the initial evaluation [20,21,24]. Responses were rated on an 11-point scale ranging from −5 (“very great deal worse”; “أسوأ بدرجة كبيرة جدا”) to +5 (“very great deal better”’; “أفضل بدرجة كبيرة جدا”), with 0 representing (“no change”; “لا يوجد أي تغير”) [24]. Participants were categorized as improved if they reported a GRC score of +2 (“Little bit better”; “أفضل بدرجة بسيطة”) or above and not improved if their GRC score was +1 (“A tiny bit better, almost the same”; “أفضل بدرجة بسيطة جدا، نفس الوضع تقريبا”) or lower. This classification is consistent with previous responsiveness studies that have used this threshold to indicate a meaningful perceived improvement [14,20]. The GRC served as an external anchor to evaluate the responsiveness of the Arabic PSFS.

### 2.5. Statistical Analysis

The responsiveness of the Arabic PSFS was evaluated based on eight a priori-defined hypotheses (Table 1) concerning the expected relationships between changes in the PSFS and changes in other outcome measures [17,25]. Following the COSMIN guideline, the PSFS was considered adequately responsive if at least 75% of the predefined hypotheses were confirmed by the data [26,27]. The distributional properties of all outcome change scores were examined through visual inspection of histograms with assessment of skewness and kurtosis statistics to assess the assumption of normality. Depending on the data distribution, Pearson’s or Spearman’s correlation coefficients were computed to test the hypothesized associations between PSFS change scores and those of the comparator measures. Pearson’s correlation coefficient was used when change scores satisfied the assumptions of approximate normality and linearity, whereas Spearman’s correlation was used when these assumptions were not met. Visual inspection of histograms together with skewness and kurtosis statistics indicated that the PSFS, RAND-36 physical functioning, RAND-36 pain, RAND-36 emotional well-being, and NPRS change scores approximated normal distributions and therefore Pearson’s correlation coefficients were used. The GRC scores demonstrated a non-normal distribution and were therefore analyzed using Spearman’s correlation coefficient. Bias-corrected and accelerated 95% bootstrap confidence intervals based on 1000 bootstrap resamples were calculated for all correlation coefficients. Statistical significance (*p*-value) for correlation analyses was obtained from the corresponding correlation tests, whereas confidence intervals were estimated using bias-corrected and accelerated bootstrap resampling; therefore, minor differences between significance tests and bootstrap confidence intervals may occur. Differences between dependent (correlated) correlations were statistically examined through the cocor online platform [28] using Meng’s test [29]. The hypotheses comparing correlations (Hypotheses 5 and 6) (Table 1) were examined based on the magnitude of the differences between dependent correlations rather than statistical significance. The study was not specifically powered for comparisons between dependent correlations using Meng’s test. All predefined responsiveness hypotheses (Table 1) were evaluated according to the prespecified correlation magnitude criterion rather than statistical significance.

A positive change score was used to represent an improvement in function or reduction in pain, while a negative change score indicated deterioration. To evaluate within-subject changes over time (baseline vs. follow-up), paired-sample *t*-tests were conducted. The magnitude of change was quantified using two distribution-based indices including Effect Size (ES) = mean change/baseline standard deviation, and Standardized Response Mean (SRM) = mean change/standard deviation of the change scores [30]. Both indices were interpreted using conventional thresholds: small (0.20–0.49), moderate (0.50–0.79), and large (≥0.80) [31]. All analyses were performed using JASP software (version 0.98.1, Amsterdam) [32].

### 2.6. Sample Size Estimation

The sample size was determined based on the COSMIN recommendations for studies evaluating responsiveness using hypothesis testing, which consider a sample of at least 50 participants to be adequate for assessing this measurement property [33]. This recommendation was used to guide study planning and should not be interpreted as a formal statistical power calculation for each individual analysis.

## 3. Results

A total of 72 participants with a broad spectrum of lower extremity musculoskeletal disorders met the inclusion criteria and were enrolled in the study (Table 2). The number of individuals screened and excluded prior to enrollment was not prospectively recorded; therefore, a complete recruitment flow before study enrollment could not be reported. Of the 72 participants, 44 (61.1%) were recruited from King Saud Medical City, 23 (31.9%) from King Fahad Medical City, and 5 (6.9%) from Physiotrio Clinic. The descriptive characteristics and summary statistics for all outcome measures at baseline and follow-up are presented in Table 3. The median interval between the two assessments was 21 days, with a range of 8 to 36 days. Participants completed a median of three treatment sessions (interquartile range: 3–4.25) before follow-up. Most participants attended one (65.3%) or two (33.3%) treatment sessions per week. Treatment consisted of individualized multimodal physical therapy, with interventions including strengthening exercises, stretching exercises, patient education, range-of-motion exercises, manual therapy, taping, dry needling, and electrotherapy in various combinations. Based on the GRC scores (median = 3; interquartile range: 2–4) obtained at the follow-up assessment, 60 participants (83.3%) reported an improvement in lower extremity function, while 12 participants (16.7%) indicated no improvement (Table 4). The distribution of PSFS scores revealed no evidence of floor or ceiling effects at baseline or follow-up (Table 3). Of the 72 participants, 44 (61.1%) nominated three activities in the PSFS, 11 (15.3%) nominated four activities, and 17 (23.6%) nominated five activities. All 72 enrolled participants completed the baseline and follow-up assessments with complete outcome data. No participants were lost to follow-up. Consequently, complete-case analyses were performed, and no imputation methods were required.

Overall, participants demonstrated significant improvement in lower extremity function and related symptoms between the two time points. The PSFS total score increased significantly from baseline to follow-up (mean difference = 1.66 points, 95% CI: 1.24–2.08, *p* < 0.001), suggesting improved lower extremity function. Similar patterns of improvement were observed in the comparator measures. The RAND-36 physical functioning scores increased significantly over time (mean difference = 8.96 points, 95% CI: 4.78 to 13.14, *p* < 0.001), reflecting reduced disability. The RAND-36 pain scores increased significantly (mean difference = 13.37 points, 95% CI: 8.14–18.60, *p* < 0.001), while the Numeric Pain Rating Scale (NPRS) scores decreased significantly (mean difference = 1.29 points, 95% CI: 0.69 to 1.89, *p* < 0.001), both reflecting a reduction in pain intensity. The magnitude of change across these measures ranged from moderate to large, with the PSFS showing a large effect among all participants and even larger effect among those classified as improved (Table 3). The RAND-36 emotional well-being scores significantly increased over time (mean difference = 4.56 points, 95% CI: 0.96 to 8.15, *p* = 0.01), reflecting a small effect (Table 3).

Table 5 presents the correlations between PSFS change scores and the changes in comparator outcome measures. The predefined responsiveness hypotheses were evaluated according to the observed correlation magnitudes relative to the prespecified thresholds. Confidence intervals and *p*-values were reported as supplementary descriptive statistics and were not used to determine whether a hypothesis was confirmed. As hypothesized, PSFS change scores showed a moderate positive correlation with the RAND-36 physical functioning domain, confirming hypothesis 1 (Table 1) and supporting the conceptual overlap between the two measures (Table 5). The correlation between PSFS and RAND-36 pain change scores was small and not significant; therefore, hypothesis 2 was not supported (Table 1 and Table 5). The PSFS change score showed a significant positive correlation with NPRS change scores, but the magnitude of the correlation was lower than hypothesized; thus, our hypothesis was not confirmed (Table 1 and Table 5).

**Figure 1:**
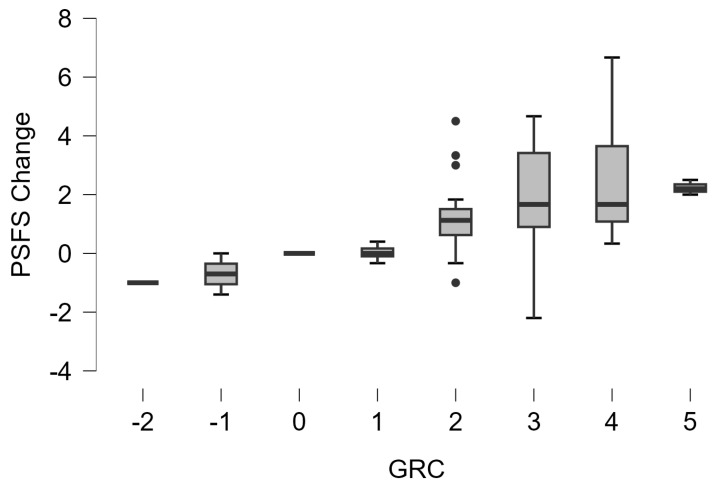
The boxplot illustrates the distribution of the Patient-Specific Functional Scale (PSFS) change scores in relation to the global rating of change scores (GRC). The number of participants in each GRC category was: −2 (*n* = 1), −1 (*n* = 2), 0 (*n* = 2), 1 (*n* = 7), 2 (*n* = 16), 3 (*n* = 23), 4 (*n* = 18), and 5 (*n* = 3).

Consistent with Hypothesis 4, the correlation between PSFS and RAND-36 Emotional Well-being was weak, as expected for distinct constructs (Table 1 and Table 5). The correlation between PSFS and RAND-36 Physical Functioning change scores was stronger than that with both RAND-36 Pain (Δ*r* = 0.31; 95% CI: 0.06 to 0.66; *p* = 0.02) and NPRS (Δ*r* = 0.22; 95% CI: −0.05 to 0.58; *p* = 0.10), confirming hypotheses 5 and 6 (Table 1 and Table 5). The PSFS change scores showed a significant positive correlation with GRC scores in line with our hypothesis 7 (Figure 1). Among participants classified as improved, PSFS demonstrated a large magnitude of change (ES and SRM ≥ 0.50), supporting our predefined hypothesis 8 (Table 3). Overall, the results supported six out of the eight (75%) predefined hypotheses.

## 4. Discussion

The present study examined the responsiveness of the Arabic version of the PSFS in individuals with lower extremity musculoskeletal disorders undergoing physical therapy. The findings demonstrate sufficient responsiveness according to the prespecified COSMIN hypothesis-testing criterion, with six of the eight predefined hypotheses (75%) confirmed. Because two hypotheses were close to their prespecified decision boundaries, replication in larger samples using lower extremity-specific comparator measures would further strengthen confidence in these findings. The present study primarily provides evidence for the ability of the Arabic PSFS to detect improvement, as only one participant reported a deterioration greater than a trivial change (GRC = −2), precluding a meaningful evaluation of responsiveness to worsening. Beyond confirming responsiveness according to COSMIN criteria, these results highlight the distinctive value of the PSFS as a patient-centered instrument that captures functional recovery through activities that are individually meaningful and contextually relevant.

The observed improvement in PSFS scores, coupled with large effect size and standardized response mean values among participants classified as improved, indicates that the Arabic PSFS detected improvement in lower extremity function over the study follow-up period. The present study evaluated responsiveness using the hypothesis-testing approach recommended by COSMIN, in which ES and SRM were incorporated as one of the predefined hypotheses alongside hypotheses examining relationships between PSFS change scores and external measures of change. Therefore, ES and SRM were not interpreted as stand-alone indicators of responsiveness but as components of the overall hypothesis-testing framework. The clinical importance of the observed 1.66-point mean change in the PSFS cannot be interpreted as clinically important without an established Arabic PSFS minimal important change threshold, which has not yet been established. Our findings are consistent with prior investigations demonstrating sufficient responsiveness of the PSFS across a wide range of musculoskeletal conditions, including lower extremity disorders, low back pain, and upper extremity conditions [12,13,14]. Notably, COSMIN-based systematic reviews have consistently rated the PSFS as having sufficient to high responsiveness when evaluated using hypothesis-testing approaches anchored to patient-perceived change [12,13,14].

Unlike fixed-item PROMs, the PSFS operationalizes function through patient-nominated activities, allowing responsiveness to be evaluated relative to outcomes that patients personally prioritize. This individualized structure might enhance sensitivity to change, as improvements are measured within tasks that are meaningful to the individual rather than against a standardized item set that may not reflect their functional goals. This characteristic aligns strongly with contemporary patient-centered and value-based care frameworks, which emphasize outcomes that reflect patient values and lived experience rather than solely clinician-defined benchmarks [3]. This patient-centered nature of the PSFS made the scale more responsive in earlier studies compared with fixed-item patient-reported outcome measures, as reported in patients with neck and back pain [34,35]. However, the present study did not formally compare the responsiveness of the PSFS with fixed-item patient-reported outcome measures.

The absence of floor and ceiling effects further supports the suitability of the PSFS for longitudinal assessment. Similar findings have been reported in PSFS validation studies across languages and anatomical regions, where individualized item generation minimizes score saturation and preserves responsiveness across the functional continuum [14]. This feature is particularly advantageous in heterogeneous lower extremity populations, where baseline function and recovery trajectories could vary substantially.

As hypothesized, PSFS change scores demonstrated a moderate positive association with changes in the RAND-36 physical functioning domain. This finding supports convergent change score validity and our hypothesis that change in lower extremity function is reflected by the observed PSFS change scores. The RAND-36 physical functioning domain assesses general physical functioning rather than lower extremity-specific function. Consequently, although the observed correlation supports the predefined hypothesis, it should be interpreted as evidence of convergent responsiveness with a generic measure of physical functioning rather than a region-specific functional instrument. Our finding is consistent with previous PSFS studies reporting moderate correlations between PSFS change scores and generic or region-specific physical function measures [14,36,37]. Importantly, the magnitude of the association suggests conceptual overlap without redundancy. This pattern is theoretically expected. While the RAND-36 physical functioning domain captures broad limitations in mobility and physical activities, the PSFS reflects change in highly specific, patient-selected tasks. As a result, improvements in individualized activities may not translate proportionally into changes across a generic functional domain. Our findings reinforce the interpretation that the PSFS provides complementary information rather than duplicating fixed-item functional measures.

While correlations between the PSFS change score and those of the RAND-36 pain domain and NPRS change scores were in the expected direction, they were weaker than hypothesized. The correlation with the NPRS approached but did not reach the anticipated strength; therefore, the pre-specified hypothesis was not confirmed. These findings suggest that the Arabic PSFS is more responsive to changes in patient-specific functional performance than to changes in pain alone, which is consistent with the intended construct measured by the PSFS. The majority of participants in the current study presented with chronic symptoms (median duration: 30 weeks). This chronicity might have contributed to the weaker-than-expected correlation between changes in pain and function, as long-standing symptoms often involve additional factors. No previous studies have established the longitudinal relationship between PSFS change scores and change in pain measures in individuals with lower extremity musculoskeletal disorders. Thus, the findings of the current study could not be directly compared to other similar studies. On the other hand, the relationship between PSFS change and change in pain has been studied and reported in other body regions [14]. In individuals with upper extremity musculoskeletal disorders, increase in PSFS scores reflecting better function was reported to be associated with reduction in pain intensity [14,38]. The same pattern of correlation has also been reported in individuals with spinal musculoskeletal disorders [14].

As expected, PSFS change scores demonstrated a weak association with changes in emotional well-being, supporting divergent change score validity. To the best of our knowledge, this is the first study in the literature that reported the association between PSFS change and mental health change over time in individuals with lower extremity musculoskeletal disorders. The pattern observed in the current study aligns with a previous report in individuals with shoulder pain showing that functional recovery and improvements in mental health have a weak association [39]. Similar weak associations between PSFS and mental health domains have been reported previously in individuals with lower extremity musculoskeletal disorders using cross-sectional data but not longitudinally using change score data [14]. Our finding reinforces the interpretation that the PSFS is primarily sensitive to improvement in physical function rather than global psychological status. While emotional well-being is an important dimension of health, it represents a construct that is theoretically distinct from task-specific functional performance. The observed correlation pattern therefore strengthens confidence in the construct validity of PSFS change scores.

The moderate association between PSFS change scores and the perceived global lower extremity functional change, measured using the GRC, provides further evidence that the Arabic PSFS captures improvement in physical function from the patient’s perspective. The GRC is widely used as an external anchor in responsiveness studies and is particularly relevant when evaluating patient-centered instruments, especially when framed to inquire about perceived change in the construct of interest that is lower extremity function in the current study [17]. Like other retrospective transition ratings, the GRC may be influenced by recall bias and participants’ current health status. Nevertheless, it remains one of the most commonly used external anchors for evaluating responsiveness. Previous PSFS investigations have consistently reported moderate correlations between PSFS change scores and global ratings of change, supporting the interpretation that PSFS-detected improvements align with patients’ subjective perceptions of recovery [14,37,38,40]. This alignment is clinically important, as outcome measures are most useful when detected changes correspond to improvements that patients themselves recognize. The present findings therefore support the PSFS as a clinically relevant tool for monitoring progress, guiding treatment decisions, and facilitating shared decision-making.

Although the GRC scale was collected as an external anchor and incorporated into the predefined hypothesis-testing approach, anchor-based responsiveness analyses, including Receiver Operating Characteristic analysis and estimation of the minimal important change, were not performed. Estimation of the minimal important change generally requires a larger sample size than that needed for responsiveness testing and is preferably based on samples with a more balanced representation of individuals who experience a clinically important change and those who do not, to minimize bias in the estimated minimal important change [41,42]. Because the present study included a relatively modest sample and the majority of participants (83%) reported improvement, the distribution of improved and not improved participants was not optimal for deriving a robust anchor-based minimal important change. Future studies should establish the minimal important change in the Arabic PSFS using larger samples with a more balanced distribution of clinical change and evaluate discriminative responsiveness using anchor-based methods. The predominance of participants reporting improvement reflects the clinical course of the study cohort and enabled evaluation of the Arabic PSFS in detecting functional improvement. The findings provide limited evidence regarding the instrument’s ability to detect deterioration. Future responsiveness studies should include participants representing a broader range of clinical outcomes, including deterioration, to more comprehensively evaluate responsiveness across the full spectrum of change.

Demonstrating responsiveness of the Arabic PSFS has important implications for musculoskeletal rehabilitation in Arabic-speaking contexts. Fixed-item PROMs may omit culturally specific or context-dependent activities that are central to patients’ daily lives. By allowing patients to nominate their own functional goals, the PSFS accommodates cultural variability and individual priorities, enhancing its relevance and acceptability. In clinical practice, the responsiveness of the PSFS supports its use for tracking individual progress and monitoring functional improvement over time. In research settings, it offers a valuable complement to standardized measures by capturing dimensions of recovery that may otherwise remain unmeasured. These attributes align closely with contemporary calls for integrating patient-reported outcomes into value-based care models [3].

This study adhered to COSMIN recommendations for responsiveness assessment through a priori hypothesis testing and the use of an external anchor, strengthening confidence in the findings. Participants received individualized multimodal physical therapy and were reassessed after varying treatment durations and numbers of treatment sessions. Although this variability may have contributed to differences in the magnitude of observed change, it reflects routine outpatient physical therapy practice, which was the intended context for evaluating responsiveness. Nevertheless, several considerations warrant attention. First, although responsiveness was established, the interpretability of PSFS change scores was not examined. Future studies should establish the minimal important change to facilitate clinical interpretation of score changes, evaluate responsiveness in individuals reporting deterioration, and confirm these findings in other Arabic-speaking countries and healthcare settings. Second, nearly two-thirds of the participants had knee/leg disorders, whereas relatively few participants had hip/thigh disorders. Consequently, the responsiveness estimates are likely to be driven primarily by knee/leg conditions and should be interpreted cautiously for less represented groups. Although the study included a broad range of lower-extremity musculoskeletal disorders representative of outpatient physical therapy practice, several diagnostic categories were represented by relatively few participants. Consequently, the responsiveness estimates should be interpreted as applying to a heterogeneous outpatient population rather than to specific diagnostic groups. Future studies with larger diagnosis-specific samples are warranted to determine whether responsiveness differs according to diagnosis, chronicity, or treatment stage. In addition, participants were recruited using convenience sampling from three outpatient physical therapy clinics, which may have introduced selection bias and may limit the generalizability of the findings to other healthcare settings or patient populations. Because the treating physical therapists administered the follow-up PSFS and had access to the baseline activity ratings, expectation or interviewer bias cannot be excluded. Although reviewing the original activities is necessary for longitudinal PSFS administration, future validation studies should consider independent or blinded follow-up administration where feasible. Finally, although the overall sample satisfied the COSMIN recommendation for responsiveness studies, the relatively small number of participants classified as not improved limited the precision of subgroup estimates. Accordingly, subgroup effect sizes should be interpreted cautiously. Future studies should further evaluate the responsiveness of the Arabic PSFS by examining its relationship with lower extremity-specific patient-reported outcome measures, such as the Arabic LEFS, and performance-based functional assessments.

## 5. Conclusions

The Arabic version of the PSFS demonstrates sufficient responsiveness in detecting improvement in lower extremity function among individuals with lower extremity musculoskeletal disorders. Its individualized, patient-centered design allows it to capture patient-specific functional changes that may not be fully reflected by pain intensity or generic health measures. These findings support the use of the Arabic PSFS as a valuable outcome measure for monitoring functional improvement in clinical practice and research among Arabic-speaking adults with lower-extremity musculoskeletal disorders receiving outpatient physical therapy. Further studies are warranted to establish its interpretability and responsiveness across specific diagnostic populations and other Arabic-speaking settings.

## Figures and Tables

**Table 1:** Predefined hypotheses for testing the responsiveness of the Arabic PSFS.

Hypothesis No.	Hypothesis Description	Expected Direction/Strength	Observed Result	Confirmed
1	Positive correlation between PSFS change scores and RAND-36 Physical Functioning change scores	*r* ≥ 0.40	0.51	Yes
2	Positive correlation between PSFS change scores and RAND-36 Pain change scores	*r* ≥ 0.30	0.20	No
3	Positive correlation between PSFS change scores and NPRS change scores	*r* ≥ 0.30	0.29	No
4	Weak correlation between PSFS change scores and RAND-36 Emotional Well-being change scores	−0.30 < *r* < 0.30	0.26	Yes
5	Correlation between PSFS and RAND-36 Physical Functioning change scores expected to be stronger (by ≥0.10) than that with RAND-36 Pain change scores	Δ*r* ≥ 0.10	0.31	Yes
6	Correlation between PSFS and RAND-36 Physical Functioning change scores expected to be stronger (by ≥0.10) than that with NPRS change scores	Δ*r* ≥ 0.10	0.22	Yes
7	Positive correlation between PSFS change scores and GRC	*r* ≥ 0.40	0.55	Yes
8	The PSFS expected to show medium or larger effect size (ES) and standardized response mean (SRM) among improved participants	ES, SRM ≥ 0.50	0.96; 1.17	Yes

PSFS = Patient-Specific Functional Scale, NPRS = Numeric Pain Rating Scale, RAND-36 = RAND-36 Item Health Survey, GRC = Global Rating of Change Scale.

**Table 2:** Characteristics of participants (*N* = 72).

Variable	Mean ± SD or *N* (%)
Age (years)	40.10 ± 11.11
Sex	
Male	29 (40.3%)
Female	43 (59.7%)
Height (m)	1.65 ± 0.10
Weight (kg)	76.50 ± 18.88
Body Mass Index (Kg/m^2^)	28.33 ± 7.22
Site of dysfunction	
Hip/Thigh	4 (5.6%)
Hip disorders (OA, Gluteal tendinopathy, hip pain)	3 (4.2%)
Postoperative fracture fixation	1 (1.4%)
Knee/Leg	45 (62.5%)
Knee OA	11 (15.3%)
Ligamentous/meniscal disorders	12 (16.7%)
Patellofemoral disorders	8 (11.1%)
Other knee disorders	12 (16.7%)
Total knee arthroplasty	2 (2.8%)
Ankle/Foot	23 (31.9%)
Plantar fasciitis	10 (13.9%)
Ligament injuries/sprains	7 (9.7%)
Other foot/ankle disorders	6 (8.3%)
Affected side	
Right	23 (31.9%)
Left	26 (36.1%)
Bilateral	23 (31.9%)
Lower extremity surgery	
Yes	6 (8.3%)
Time after surgery (weeks)	15.0 (37.8) *
No	66 (91.7%)
Duration of symptoms (weeks)	30.0 (56.5) *

* = median (interquartile range); OA = Osteoarthritis.

**Table 3:** Outcome measures at baseline and follow-up (*N* = 72).

Variable	N	Baseline Scores Mean ± SD	Follow-Up Scores Mean ± SD	Change Scores Mean ± SD	ES (95% CI)	SRM (95% CI)	Baseline		Follow-Up	
							Floor	Ceiling	Floor	Ceiling
PSFS (0–10)	72	4.32 ± 2.08	5.97 ± 2.18	1.66 ± 1.80	0.80 (0.57–1.03)	0.92 (0.64–1.2)	2.8%	0%	0%	1.4%
Improved	60	4.39 ± 2.11	6.42 ± 1.93	2.03 ± 1.74	0.96 (0.68–1.24)	1.17 (0.84–1.49)				
Not improved	12	3.95 ± 1.99	3.77 ± 2.11	−0.18 ± 0.52	−0.09 (−0.23–0.05)	−0.35 (−0.93–0.24)				
RAND-36 physical functioning (0–100)	72	47.71 ± 23.04	56.67 ± 24.57	8.96 ± 17.80	0.39 (0.21–0.57)	0.50 (0.26–0.75)	4.2%	1.4%	1.4%	2.8%
RAND-36 emotional well-being (0–100)	72	67.67 ± 17.90	72.22 ± 17.44	4.56 ± 15.31	0.25 (0.05–0.45)	0.30 (0.06–0.53)	0%	2.8%	0%	4.2%
RAND-36 pain (0–100)	72	47.54 ± 22.74	60.90 ± 23.07	13.37 ± 22.25	0.59 (0.35–0.83)	0.60 (0.35–0.85)	2.8%	4.2%	1.4%	4.2%
NPRS (0–10)	72	5.49 ± 2.50	4.19 ± 2.58	1.29 ± 2.55	0.52 (0.27–0.77)	0.51 (0.26–0.75)	2.8%	2.8%	1.4%	9.7%

PSFS = Patient-Specific Functional Scale, RAND-36 = RAND-36 Item Health Survey, NPRS = Numeric Pain Rating Scale, ES = Effect Size, SRM = Standardized Response Mean, SD = Standard Deviation, CI = Confidence Interval. Paired-test results and mean-change confidence intervals are presented within the text of the Results section.

**Table 4:** Participants according to their global rating of change score at follow-up.

Variable	*N* (%)
GRC	
5 (Very great deal better)	3 (4.2)
4 (Great deal better)	18 (25.0)
3 (Moderately better)	23 (31.9)
2 (Little bit better)	16 (22.2)
1 (A tiny bit better, almost the same)	7 (9.7)
0 (No change)	2 (2.8)
−1 (Tiny bit worse, almost the same)	2 (2.8)
−2 (Little bit worse)	1 (1.4)
−3 (Moderately worse)	0 (0.0)
−4 (Great deal worse)	0 (0.0)
−5 (Very great deal worse)	0 (0.0)
Improved vs Not improved	
Improved (GRC scores 2, 3, 4, 5)	60 (83.3%)
Not improved (GRC scores −5, −4, −3, −2, −1, 0, 1)	12 (16.7%)

GRC = Global Rating of Change Scale.

**Table 5:** Correlation between the PSFS change score and change in other measures.

Variables	r (95% CI)	*p* Value
RAND-36 physical functioning	0.51 (0.29 to 0.66)	<0.001
RAND-36 pain	0.20 (−0.11 to 0.47)	0.09
RAND-36 emotional well-being	0.26 (−0.05 to 0.51)	0.03
NPRS	0.29 (0.07 to 0.47)	0.02
GRC	0.55 (0.35 to 0.70) *	<0.001

r = Pearson’s correlation coefficient; * = Spearman’s correlation coefficient; CI = Confidence interval; PSFS = Patient-Specific Functional Scale, RAND-36 = RAND-36 Item Health Survey; NPRS = Numeric Pain Rating Scale, GRC = Global Rating of Change Scale.

## Data Availability

The data that support the findings of this study are available from the corresponding author, upon reasonable request.
